# Ellagic Acid and Gut Microbiota: Interactions, and Implications for Health

**DOI:** 10.1002/fsn3.70133

**Published:** 2025-04-06

**Authors:** Pinze Leng, Ye Wang, Minhao Xie

**Affiliations:** ^1^ School of Medicine Jiangsu University Zhenjiang China; ^2^ Collaborative Innovation Center for Modern Grain Circulation and Safety, College of Food Science and Engineering Nanjing University of Finance and Economics Nanjing China; ^3^ Jiangsu Province Engineering Research Center of Edible Fungus Preservation and Intensive Processing Nanjing China

**Keywords:** ellagic acid, gut microbiota, health benefits, interactions, urolithins

## Abstract

Ellagic acid (EA), a widely distributed natural polyphenolic acid existing in many kinds of plant‐based foods, undergoes complex physical and chemical transformations during digestion and biotransformation. Particularly, EA is metabolized by gut microbiota and transformed into urolithins in the colon. These metabolites exhibit enhanced bioavailability and bioactivity. This review explores the intricate interactions between EA and gut microbiota, emphasizing their implications for human health. We discuss the role of gut microbiota in EA metabolism, resulting in distinct metabolic phenotypes associated with varying urolithin production profiles. EA and its gut‐derived metabolites, urolithins, have been reported to have the potential to modulate the microbial community composition and function of gut microbiota, promoting beneficial bacteria while reducing harmful ones. Furthermore, EA and urolithins exhibit a spectrum of beneficial biological activities, including antioxidant, anti‐inflammatory, and anticancer properties, along with enhancements to intestinal barrier function and modulatory effects on metabolic and cardiovascular systems, through molecular mechanisms such as activating Nrf2 and inhibiting NF‐κB pathways. The review highlights and compares the potential of EA and its gut microbial metabolites in the prevention and treatment of various diseases. However, further studies are required to elucidate the underlying mechanisms of the interactions between EA and gut microbiota and their health benefits. Continued investigation into EA and its metabolites is essential for advancing our understanding of their role in promoting human health and developing novel therapeutic applications.

## Graphical abstract

The review discusses the interactions between ellagic acid (EA) and gut microbiota and highlights the potential of EA and its gut microbial metabolites in bioactivity.

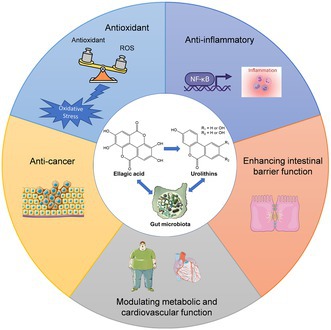

## 1 Introduction

Ellagic acid (EA, 2,3,7,8‐tetrahydroxy‐[1]benzopyrano[5,4,3‐*cde*][1]benzopyran‐5,10‐dione, C_14_H_6_O_8_, Molar mass: 302.194 g/mol) is a naturally occurring polyphenolic compound widely distributed in various plant‐based foods, such as raspberries (~1.5 mg/g dry weight), walnuts (~0.6 mg/g), strawberries (~0.6 mg/g dry weight), pecans (~0.3 mg/g), and cranberries (~0.1 mg/g) (Ríos et al. 2018). EA exists in the forms of its precursor ellagitannins and EA derivatives as well, including punicalagin and granatins. The levels of total EA derivatives could reach up to ~29 mg/g and ~182 mg/g dry weight in ripe and thinning pomegranate fruits, respectively (Nuncio‐Jáuregui et al. 2015). Its unique chemical structure, comprising two lactones, four hydroxyl groups and two hydrocarbon rings, contributes to its redox potential and antioxidant activity (Ghosh et al. 2021). However, the low water solubility of EA at near‐body temperature (9.7 μg/mL, 37°C) poses challenges to its bioavailability, making its effective utilization a key focus of current research.

Ellagic acid has significant ethnopharmacological relevance and is employed in traditional medicine for the prevention and treatment of various diseases. For example, pomegranate, rich in EA, is utilized in some traditional medical systems for its antidiabetic, anti‐inflammatory, and wound‐healing properties (Les et al. 2018). In addition, in the Amazon rainforest, decoctions and teas containing EA are employed for wound healing and inflammation treatment, underscoring its diverse therapeutic potential (Esposito et al. 2023). These traditional uses indicate that EA has substantial health‐benefiting effects on metabolic, inflammatory, and oxidative stress‐related conditions.

The intestinal microbiota, a complex collection of microorganisms inhabiting the gastrointestinal tract, has the formidable capacity to digest food components, biosynthesize essential vitamins, eliminate pathogens, stimulate and regulate the host immune system, detoxify and remove carcinogens, and support intestinal functions. Under normal conditions, the gut microbiota maintains a dynamic equilibrium of the ecosystem with the external environment and the host. Once the microecological homeostasis is disrupted, it can result in multiple host dysfunctions, including inflammation, loss of barrier function, and immune dysfunction, potentially inducing diseases (Zhai et al. 2020). Recent research into the metabolic role of gut microbiota has revealed that it participates in the metabolism of EA through various pathways, affecting its in vivo metabolic process and efficacy. Therefore, studying the impacts of intestinal microbiota on EA metabolism and its metabolites is of great significance.

Emerging studies reveal that gut microbiota significantly influence the metabolism and bioactivity of EA. During the digestion and biotransformation of EA, a series of complex physical and chemical changes occur. Ellagitannins, the primary form of EA in foods, are hydrolyzed into EA during digestion in the upper digestive tract. The human gut microbiota and the host form a co‐metabolic structure, participating in EA's metabolic process. From a chemical structure perspective, gut microbiota such as *Enterocloster bolteae* and *Parabacteroides gordonii* biotransform EA into metabolites urolithins through a series of reactions, including cleavage of lactone ring, decarboxylation, and dehydroxylation. These metabolites exhibit improved bioavailability compared to EA, the metabolites urolithins are more readily absorbed in the body, where they conjugate with glucuronic acid and enter systemic circulation to reach target organs. The intestinal microbiota plays a pivotal role in the alteration of EA's composition and properties. Interestingly, EA itself modifies the composition of intestinal microbiota by reducing harmful bacteria and increasing beneficial ones, thus affecting the intestinal microbial community and highlighting a bidirectional interaction between EA and the intestinal microbiome. Identifying the intestinal microbial species involved in the transformation of EA, elucidating the interactions between EA and intestinal microbiota, and understanding their metabolic pathways are crucial for elucidating the mechanisms underlying EA's health benefits.

Ellagic acid and its metabolites have already been extensively investigated for their broad health benefits. EA and its metabolites share key health‐benefiting effects, including anti‐inflammatory, antioxidant, and antitumor capacities, though their mechanisms of action may differ. Beyond these traditional pharmacological roles, recent experiments suggest broader applications for EA in health promotion (Ríos et al. 2018). This review aims to provide a comprehensive overview of the interactions between EA and gut microbiota, focusing on the biotransformation of EA, the health implications of its metabolites, and the bidirectional influence between EA and intestinal microbes. Finally, we discuss current research gaps, challenges, and future prospects to advance the development of EA‐based therapeutics.

## 2 Metabolism of EA in Gastrointestinal Tract

The bioavailability and health‐benefiting activities of EA are significantly affected by its metabolism in the digestive tract, particularly by the gut microbiota. The human digestive tract harbors diverse and complex microbial communities that play a crucial role in the metabolism of dietary compounds, including EA. Upon ingestion, EA, derived from the hydrolysis of ellagitannins in the stomach and small intestine, undergoes extensive biotransformation mediated by intestinal microorganisms, resulting in the production of various metabolites, primarily urolithins. In addition to microbial metabolism, EA is also converted into EA glucuronide and EA sulfate in the liver and intestines (Espín et al. 2013). However, these conjugates are not the primary metabolites responsible for the bioactivities of EA. The primary biotransformation of EA occurs in the lower digestive tract, where specific gut bacteria catalyze its conversion into urolithins. These metabolites are considered the key bioactive forms of EA due to their superior bioavailability and enhanced biological activities compared to the parent compound.

### 2.1 Metabolic Pathways of EA

**FIGURE 1:**
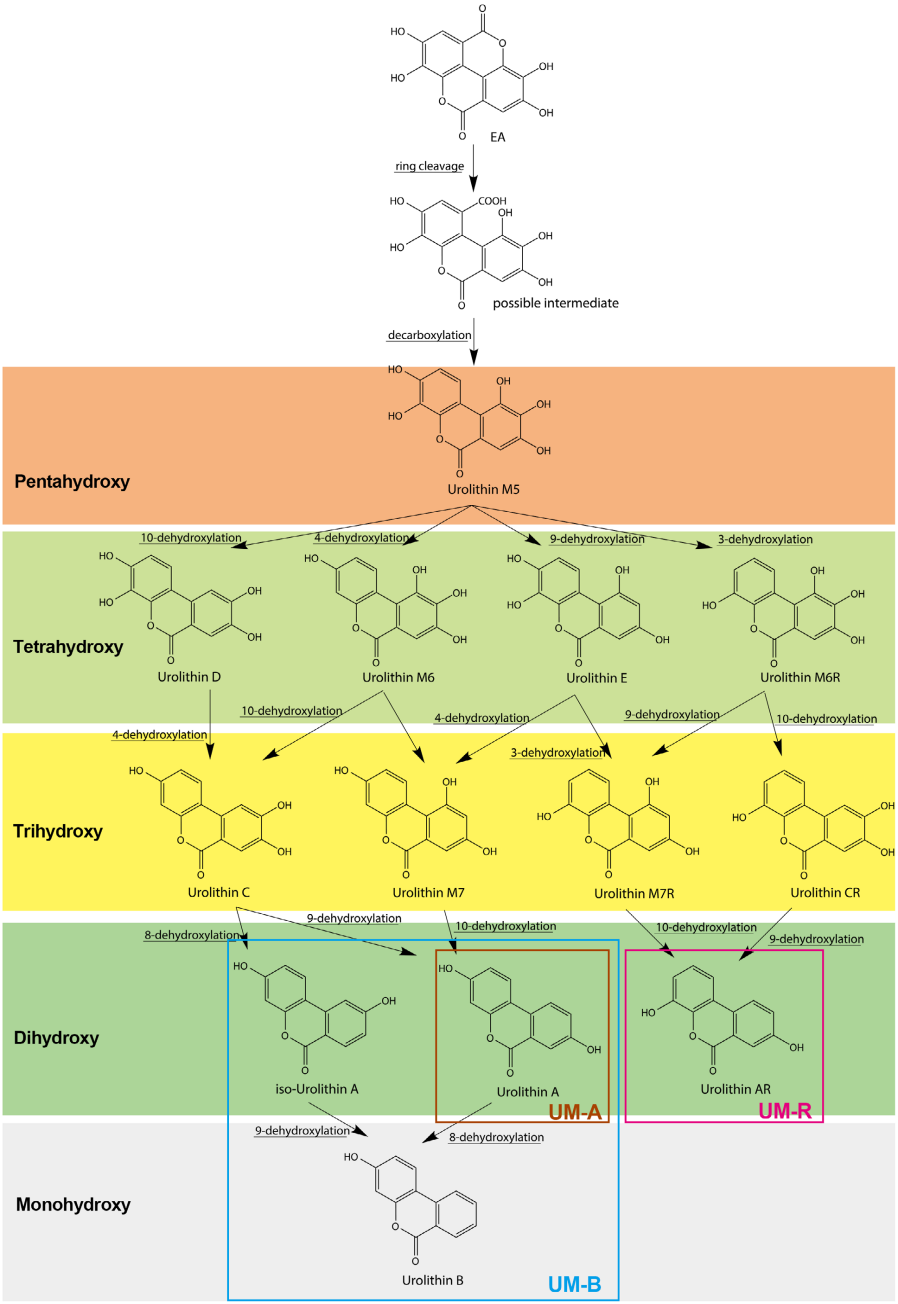
Proposed transformation pathways of ellagic acid into urolithins by intestinal microbiota.

The biotransformation of EA into urolithins involves a series of enzymatic reactions catalyzed by intestinal bacteria. These conversions are influenced by the community composition and functional profile of an individual's intestinal microbiota, resulting in personalized metabolic pathways due to the distinct inhabiting microbiome, which will be further discussed in Section 2.2. The proposed overall metabolic pathways of EA are depicted in Figure 1 (Hua et al. 2024). In the colonic environment, EA metabolism begins with lactone ring hydrolysis and decarboxylation, resulting in the generation of urolithin M5, a pentahydroxy compound. This compound subsequently undergoes a series of dehydroxylation transformations, where a hydroxyl group at various positions is removed to yield the tetrahydroxy urolithins, including urolithin D, urolithin E, urolithin M6, and urolithin M6R. The removal of another hydroxyl group is involved in further metabolism, contributing to the production of trihydroxy‐urolithins, namely urolithin C, urolithin G, urolithin M7, urolithin M7R, urolithin CR, and iso‐urolithin G. Subsequently, dihydroxy‐urolithins are generated, such as urolithin A, urolithin AR, and iso‐urolithin A. The metabolic pathway concludes with the removal of one hydroxyl group from iso‐urolithin A (or urolithin A), producing monohydroxy urolithins, specifically urolithin B. These metabolic transformations highlight the intricate bioconversion of EA and emphasize the potential biological functions and mechanisms of action of these urolithin metabolites. Urolithins, as the primary gut microbiota‐derived metabolites of EA, are considered pivotal in mediating its health‐promoting effects.

During EA biotransformation, intestinal microbiota plays pivotal roles in three kinds of conversions: lactone hydrolysis, decarboxylation, and dehydroxylation. Among these, pyrocatechol‐dehydroxylase is the primary enzyme involved. Previous studies demonstrate that pyrocatechol‐dehydroxylases exhibit diversity within the gut microbiota. These enzymes are characterized by confined substrate spectrums, suggesting that the observed variety in the dehydroxylation stages can be attributed to the presence of various pyrocatechol‐dehydroxylases (Rekdal et al. 2020). These enzymes not only catalyze crucial steps in the metabolic pathway of EA but also contribute to the complexity and diversity of this process within the colonic environment. Despite these insights, the complete enzymatic processes involved in EA biotransformation remain poorly understood. Many intermediate metabolites have yet to be identified, and further research is required to elucidate the specific enzymatic mechanisms underlying these transformations.

### 2.2 Role of Intestinal Microbiota in EA Metabolism

**FIGURE 2:**
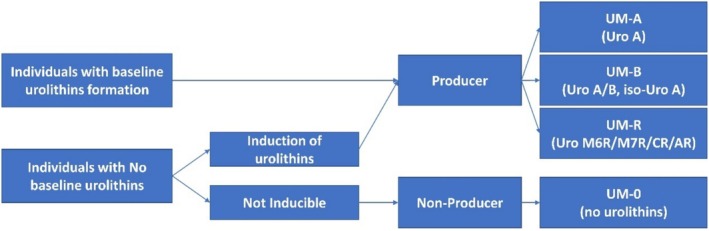
Metabolic phenotypes and their criteria of ellagic acid metabolism in individuals.

Though the intestinal microbiome encodes a considerable array of metabolic genes and enzymes, not all individuals possess the microbiota responsible for EA transformation. Depending on their ability to produce urolithins, the individuals can be categorized into distinct metabolic phenotypes, namely producers and non‐producers. Producers refer to those capable of generating urolithins during the metabolic process, while non‐producers generate only very low concentrations of urolithins (Li et al. 2015). Based on the urolithin metabolism by the intestinal microbiota, these metabolic phenotypes can be further classified into urolithin metabolic phenotype A (UM‐A), metabolic phenotype B (UM‐B), and metabolic phenotype 0 (UM‐0). UM‐A is characterized by the exclusive production of urolithin A, and UM‐B is distinguished by the production of iso‐urolithin A and/or urolithin B, along with urolithin A. In contrast, individuals with the UM‐0 phenotype do not produce detectable levels of these urolithins (Figure 2). The three urolithin metabolic phenotypes were observed irrespective of the individuals' demographic features and health status, and variations in intestinal microbiota could be associated with these phenotypes (Tomás‐Barberán et al. 2014). Besides the three main phenotypes, a new metabolic phenotype, UM‐R, has been recently identified. This phenotype produces urolithin M6R, urolithin M7R, urolithin AR, and urolithin CR (García‐Villalba et al. 2019; Zhang, Cui, Mao, Zhang, Zhao, Tang, et al. 2023; Zhang, Cui, Mao, Zhang, Zhao, Zhang, et al. 2023). The variation in production of urolithins among individuals is attributed to differences in the composition of their intestinal microbiota. Various factors, including diet, age, gender, and genetic background, can significantly influence the composition and activity of intestinal microbiota, consequently shaping the destiny of EA metabolism. Therefore, the production of urolithins and their subsequent bioactivities may differ among individuals. In addition, food matrix structures have effects on the biotransformation of EA due to increasing the solubility and dispersity (Li, Jia, et al. 2023).

The biotransformation of EA to urolithins is facilitated by specific bacterial species within the intestinal microbiota. Studies have identified several bacterial strains capable of metabolizing EA, including members of the Eggerthellaceae, Streptococcaceae, Enterococcaceae, Bifidobacteriaceae, and Lactobacillaceae families (Table 1). These bacteria, most of which have been isolated from human feces, possess the necessary enzymes to catalyze the bioconversion of EA to urolithins, including urolithin A, urolithin B, iso‐urolithin A, and urolithin C.

**TABLE 1:** Effects of ellagic acid and derived urolithins on intestinal microbiota and health benefits.

Supplement	Study model	Dose	Duration	Effect on microbial profile	Effect on SCFAs	Health impact	Reference
Pomegranate extract (6.8% EA)	Healthy volunteer	1000 mg	4 weeks	In UA producers, Actinobacteria ↑, Firmicutes ↓, *Butyrivibrio* ↑, *Enterobacter* ↑, *Escherichia* ↑, *Lactobacillus* ↑, *Prevotella* ↑, *Serratia* ↑, *Veillonella* ↑, *Collinsella* ↓	—	—	Li et al. (2015)
EA	C57BL/6J mice	1 g, 3 g EA/kg diet	21 days	*Escherichia coli* ↓ *Lactobacillus* spp. ↑	—	Enhanced growth, promoted intestinal development, increased antioxidant capacity	Xu et al. (2021)
EA	HFD‐fed C57BL/6J mice	0.3 g EA/kg diet (~30 mg/kg bw/day)	10 weeks	No significant effect on the Firmicutes/Bacteroides ratio	No significant effect on SCFAs	Reduced body weight and blood lipid	Jin et al. (2023)
EA	DSS‐induced C57BL/6 mice	100 mg/kg	6 days	Alpha‐diversity ↑, Staphylococcaceae ↓, Lactobacillaceae ↑, Rikenellaceae ↓, Staphylococcus ↓	—	Attenuated DSS‐induced ulcerative colitis	Chen et al. (2023)
EA	DSS‐induced BALB/c mice	60, 120 mg/kg bw	39 days	Bacteroidetes ↑, *Bacteroides* ↑, Muribaculaceae ↑, *Dubosiella*.↑, Firmicutes ↓, *Odoribacter* ↓, *Helicobacter* ↓, Lachnospiraceae_NK4A136_group ↓	—	Alleviated the colonic inflammation phenotype	Li, Xu, et al. (2023)
EA	Binge alcohol‐induced C57BL/6J mice	60 mg/kg bw	14 days (pretreatment)	*Bacteroides* ↑, *E. coli* ↑, *Lactobacillus* ↑	—	Prevented binge alcohol‐induced leaky gut and liver injury	Kim et al. (2021)
EA	Weaned piglets (Landrace × Yorkshire)	500 g/t diet	40 days	In the cecum, *Megasphaera* ↑, *Megasphaera elsdenii*_14_14 ↑, *Lactobacillus delbrueckii* ↑, *Lactobacillus amylovorus* ↑ In the rectum, *Lactobacillus reuteri* ↑, *Nagativibacillus* ↑, *Blautia* ↑, *Microtrichales* ↑, *Acidimicrobiia* ↑, *Catenisphaera* ↑	—	Affected the gut immune response, promoted beneficial gut microbiota, reduced inflammatory responses, and promoted the growth and intestinal health of piglets	Lu et al. (2022)
EA	Weaned piglets (Landrace × Yorkshire)	1 g/kg diet	14 days	Alpha diversity↑, Tenericutes ↑, Ruminococcaceae ↑, *Clostridium ramosum* ↑, *Parabacteroides* ↓	In colon: acetic acid ↑, propionic acid ↑, butyric acid ↑, total SCFA ↑ In jejunal: acetic acid ↑, butyric acid ↑, total SCFA ↑	Attenuated intestinal damage and oxidative stress, improved diarrhea and growth performance	Qin et al. (2022)
EA	Heat‐Stressed Arbor Acres broilers	75, 150, 300, and 600 mg/kg diet	4 weeks	*Streptococcus* ↓, *Ruminococcus_torques* ↓, *Rothia* ↓, *Neisseria* ↓, *Actinomyces* ↓, *Lautropia* ↓		Improved antioxidant capacity and the intestinal barrier function of heat‐stressed broilers	Yang et al. (2022)
EA	*Clostridium perfringens* ‐induced subclinical necrotic enteritis (SNE) Arbor Acres broilers	500 mg/kg diet	42 days	Desulfovibrionia ↑, Elusimicrobium ↑, Proteobacteria ↑, Gammaproteobacteria ↑, Enterobacteriaceae ↑, *Escherichia‐Shigella* ↑, Muribaculaceae ↑, Lachnospiraceae NK4A136 ↑, *Ruminococcus* ↓, *Fusobacterium* ↓, Fusobacteriales ↓, Fusobacteria ↓, Fusobacteriaceae ↓, Fusobacteriota ↓, Chloroflexi ↓, Intestinimonas ↓, Subdoligranulum ↓, *Lactobacillus* ↓, Lactobacillaceae ↓, Lactobacillales ↓, Bacilli ↓, Clostridia ↓, Firmicute ↓	—	Ameliorated *C. perfringens* ‐induced SNE in broilers, protect and enhance growth performance of broilers	Tang et al. (2022)
Urolithin A	C57BL/6 mice	20 mg/kg or 100 mg/kg, twice daily	7 days	Family S24‐7 ↑, *Ruminococcus* ↑, *Prevotella* ↑, *Allobaculum* ↓	Propionate ↑	Increased the mucus layer thickness and MUC2 protein level in mucosal epithelium, reduced colonic mucosal permeability	Yasuda et al. (2024)
Urolithin A	Clinical trial, participants (non‐UA producers) had relatively poor vascular endothelial function (VEF)	10, 50 mg/day	12 weeks	Alpha diversity ↑, *Escherichia‐Shigella* ↓, *Eggerthella* ↑	Propionic acid ↓	Improved flow‐mediated vasodilatation (FMD) scores in Participants having poor baseline FMD values and low Bacillota/Bacteroidota ratio	Nishimoto et al. (2023)
Urolithin A	High‐fat diet/streptozotocin‐induced diabetes mellitus C57BL/6J mice	200 mg/kg bw	10 weeks	No significant effect on beta‐diversity or Firmicutes/Bacteroides ratio	No significant effect on SCFAs	Attenuated diabetes‐associated cognitive impairment	Xiao et al. (2022)
Urolithin A	Sleep‐deprived C57BL/6 mice	50 mg/kg bw	1 week	Clostridia_UCG‐014 ↓, Candidatus_Saccharimonas ↓, *Lactobacillus* ↑, Muribaculaceae ↑	—	Enhanced muscle strength and muscular endurance	Zhu et al. (2024)
Urolithin A	Alcohol‐related liver disease (ALD) C57BL/6J mice	200 mg/kg/day	14 weeks	*Bacteroides sartorii* ↑, *Parabacteroides distasonis* ↑, *Akkermansia muciniphila* ↑	Propionic acid ↑	Ameliorated alcohol‐induced metabolic disorders and hepatic endoplasmic reticulum stress	Zhang et al. (2024)
Urolithin A	*Helicobacter pylori* ‐induced Balb/c mice	50 mg/kg/2 days	4 weeks	Firmicutes ↓, Bacteroidota ↑, Verrucomicrobiota ↑, Marinifilaceae ↓, Desulfovibrionaceae ↓, Peptococcaceae ↑, Helicobacteraceae ↓	—	Attenuated inflammation, reduced secretion of *H. pylori* virulence factors and tissue injuries	Yu et al. (2022)
Urolithin B	D‐galactose‐induced aging C57 mice	50, 100, 150 mg/kg	8 weeks	Firmicutes/Bacteroidetes ↓, Firmicutes ↓, Bacteroidetes↑, *Helicobacter* ↓, *Parasutterella* ↓, *Roseburia* ↑, *Faecalibacterium* ↑	—	Weakened small intestine injury, ameliorated intestinal immunity function	Chen et al. (2021)
Urolithin C	Choline‐deficient amino acid‐defined high‐fat diet (CDAHFD) induced NAFLD C57BL/6J mice	40 mg/kg/day	4 weeks	Firmicutes/Bacteroidota ↓, *Parabacteroides goldsteinii* ↑, *Lactobacillus vaginalis* ↑	—	Protected hepatic damage	Xu et al. (2023)

Abbreviations: “—”, Not reported; bw, body weight; EA, Ellagic acid; SCFAs, Short‐chain fatty acids.

Although the specific enzymes involved in EA biotransformation remain incompletely characterized, it is hypothesized that they are part of the bacterial metabolic pathways dedicated to the degradation of complex polyphenols. In addition to enzymatic reactions, microbial interactions also play a vital in EA metabolism. Certain intestinal bacterial species may collaborate or compete for substrates and nutrients, influencing the metabolism of EA and the production of urolithins. Some bacterial species may produce metabolites that can be utilized by other bacteria, promoting the biotransformation of EA. For example, though some intestinal microbes, such as some *Enterocloster* species, are unable to directly metabolize EA into urolithins, they could notably catalyze certain urolithin intermediates, including urolithin C, urolithin D, urolithin M6, and iso‐urolithin A, into subsequent urolithin metabolites (Iglesias‐Aguirre et al. 2023).

The role of intestinal microbiota in EA metabolism extends beyond simple biotransformation. By converting EA into urolithins, the microbiota enhances the bioavailability and potential biological activity of these substances. Furthermore, the diversity and composition of the intestinal microbiota influence both the type and quantity of urolithins produced, highlighting the importance of microbial diversity in modulating EA metabolism and its associated health benefits. Oral supplementation of an EA‐converting strain, *Gordonibacter urolithinfaciens* DSM 27213, had little impact on the local gut microbiota, and co‐administration of *G. urolithinfaciens* with EA in a synbiotic formulation significantly promoted the growth of *Akkermansia*, *Lactobacillus*, and *Bifidobacterium*, beneficial bacteria known to be associated with gut health. The synbiotic formulation remarkably enhanced the bioavailability of urolithins and increased total urolithin excretion in the urine, particularly urolithin A, compared to EA administration alone (Yang et al. 2024). In summary, the intestinal microbiota is vital in the metabolism of EA, transforming it to biologically active urolithins through a sequence of enzymatic processes. The production of these urolithin metabolites not only improves the bioavailability of EA but also provides a foundation for their various biological activities in the body. Understanding the intricate biotransformation processes involved in EA metabolism by gut microbiota is necessary for harnessing the full potential of this natural compound in nutritional and pharmaceutical applications.

## 3 Effects of EA and Its Intestinal Derivates on Intestinal Microbiota

The interaction between EA, its urolithin metabolites, and the intestinal microbiota holds critical importance in understanding their health‐promoting properties. These interactions influence the biotransformation of EA into urolithins, compounds with enhanced bioavailability and bioactivity. Investigating these effects not only elucidates their potential anti‐inflammatory, antioxidant, and antiproliferative roles but also offers insights for developing novel probiotics, prebiotics, or therapeutic strategies to improve gut health and overall well‐being. A deeper understanding of these processes could optimize the health benefits of EA and its metabolites, enhancing their application in disease prevention.

Ellagic acid has attracted considerable interest for its potential health benefits in modulating the composition and function of intestinal microbiota. The recent research findings on the impact of EA and derived urolithins on intestinal microbiota are summarized in Table 2. It has been demonstrated that the introduction of EA leads to induced changes in the makeup of the gut microbiome. In a study involving heat‐stressed broiler chickens, the supplementation of EA increased the relative abundance of *Lactobacillus* and *Ruminococcus torques*, while decreasing the abundance of *Streptococcus*, *Rothia*, and Actinomyces (Yang et al. 2022). Similarly, in a DSS‐induced mouse model of colitis, EA supplementation led to an elevation in the relative abundance of Bacteroidetes and the Muribaculaceae family, with a concurrent reduction in the proportion of Firmicutes (Li, Xu, et al. 2023). Furthermore, in weanling piglets, EA supplementation resulted in an increased abundance of Ruminococcaceae and *Clostridium ramosum* , and a decreased abundance of *Parabacteroides* (Qin et al. 2022). These findings suggest that EA can selectively modulate the intestinal microbiota, promoting the proliferation of advantageous bacteria while suppressing the growth of potentially detrimental ones.

**TABLE 2:** Bacterial strains capable of transforming ellagic acid into urolithins.

Family	Species/stain	Source	Transformation	Intermediate	Reference
Eggerthellaceae	*Gordonibacter urolithinfaciens* CEBAS 1/15P (= DSM 27213^T^ = CCUG 64261^T^)	Human feces	EA → urolithin C	Urolithin M‐5, urolithin M‐6	Selma et al. (2014)
Eggerthellaceae	*Gordonibacter pamelaeae* DSM 19378^T^	Human colon	EA → urolithin C	Urolithin M‐5, urolithin M‐6	
Eggerthellaceae	*Gordonibacter faecis* KGMB12511^T^ (= KCTC 25343^T^ = NBRC 116190^T^)	Human feces	EA → urolithin C	Urolithin M‐5, urolithin M‐6	Kim et al. (2024)
Eggerthellaceae	*Ellagibacter isourolithinifaciens* CEBAS 4A4 (= DSM 104140^T^ = CCUG 70284^T^)	Human feces	EA → isourolithin A	Urolithin M‐5, urolithin M‐6, urolithin C	Selma et al. (2017), Iglesias‐Aguirre et al. (2023)
Streptococcaceae	*Lactococcus garvieae* FUA009	Human feces	EA → urolithin A	Suspected urolithin E	Mi et al. (2022)
Streptococcaceae	*Streptococcus thermophilus* FUA329	Human breast milk	EA → urolithin A	—	Liu, Liu, et al. (2022)
Bifidobacteriaceae	*Bifidobacterium pseudocatenulatum* INIA P815	Human feces	EA → urolithin A, B	—	Gaya et al. (2018)
Enterococcaceae	*Enterococcus faecium* FUA027	Human feces	EA → urolithin A	Urolithin C	Zhang et al. (2022)
Lactobacillaceae	*Lactiplantibacillus plantarum* CCFM1286 *Lactiplantibacillus plantarum* CCFM1290 *Lactiplantibacillus plantarum* CCFM1291	—	EA → urolithin A	—	Zhang, Cui, Mao, Zhang, Zhao, Tang, et al. (2023), Zhang, Cui, Mao, Zhang, Zhao, Zhang, et al. (2023)

Abbreviations: “—”, Not reported; EA, Ellagic acid; ^T^, type strain.

Ellagic acid not only affects the composition of intestinal microbiota but also influences their functional profile. In heat‐stressed broiler chickens, functional prediction analysis of the intestinal microbiota revealed that there were enrichments in functions related to lipid and carbohydrate metabolism in the EA‐treated group (Yang et al. 2022). In DSS‐induced colitis mice, functional prediction of the intestinal microbiota in the EA‐supplemented group showed activation of pathways related to tight junctions, neuroactive ligand‐receptor interaction, and peroxisome proliferator‐activated receptor (PPAR) signaling (Li, Xu, et al. 2023). Additionally, in weanling piglets, the intestinal microbiota functional prediction in the EA‐supplemented group indicated enrichment of pathways related to carbohydrate metabolism, glycoside biosynthesis and metabolism, and the digestive system (Qin et al. 2022). These results suggest that EA can modulate the functional capacity of the intestinal microbiota, potentially contributing to improved host health.

The modulation of intestinal microbiota by EA has been linked to positive effects on host intestinal health. In heat‐stressed broiler chickens, EA supplementation improved antioxidant capacity and intestinal barrier function (Yang et al. 2022). In the DSS‐induced colitis model, EA supplementation alleviated colitis symptoms and improved intestinal health through the regulation of the expression of tight junctions and PPAR signaling‐related genes (Li, Xu, et al. 2023). Furthermore, in weanling piglets, EA supplementation improved intestinal morphology, enhanced antioxidant capacity, and promoted intestinal health by modulating the makeup of the intestinal microbiome (Qin et al. 2022). These results indicate that the beneficial effects of EA on host health are mediated, at least partially, through its modulation of the intestinal microbiota.

Urolithin A, an intestinal microbiota‐produced metabolite from EA, exerts significant effects on the intestinal microbiota, modulating both microbial community composition and functional profiles. The consumption of urolithin A has been demonstrated to modify the intestinal microbial ecosystem, resulting in alterations that can contribute to various health benefits. Several research studies have explored the effects of urolithin A on the intestinal microbiome, revealing a complex interplay between this microbial metabolite and the gut microbial community. One notable effect of urolithin A on the intestinal microbiota is the alteration of microbial community composition. In a study by Nishimoto et al. (2023), the consumption of urolithin A was linked to an enhancement in alpha diversity of the gut microbiota, as indicated by higher Faith's phylogenetic diversity. Furthermore, specific changes in microbial genera were observed, with 4 and 9 genera significantly changed in participants receiving a dose of 10 mg and 50 mg of urolithin A per day, respectively. These findings suggest that urolithin A can shape the intestinal microbial community by enhancing the proliferation of certain microbial species and suppressing others.

In addition to compositional changes, urolithin A also influences the functional profiles of the intestinal microbiota. The intestinal microbiota plays a pivotal role in metabolism, immune responses, and the maintenance of intestinal barrier integrity, among other functions. Urolithin A has been shown to promote the generation of short‐chain fatty acids (SCFAs), particularly propionic acid, which are beneficial metabolites produced by gut bacteria (Zhang et al. 2024). Increased levels of SCFAs have been linked to improved intestinal health, boosted immune responses, and reduced inflammation. Furthermore, urolithin A has been documented to enhance the expression of tight junction proteins, including Occludin and ZO‐1, that are essential for maintaining the integrity of the intestinal barrier (Singh et al. 2019). This effect of urolithin A on tight junction proteins is likely mediated through its interactions with intestinal microbiota, as it plays a significant role in regulating intestinal permeability.

Moreover, urolithin A has been investigated for its health benefit potentials depending on its effects on the gut microbiota, such as alleviating alcohol‐related liver disease (ALD). In the study conducted by Zhang et al. (2024), urolithin A was found to restore ALD‐induced gut microbiota dysbiosis by enriching the levels of beneficial bacteria such as *Parabacteroides distasonis* , *Bacteroides sartorii* , and *Akkermansia muciniphila* . These bacteria are known to produce SCFAs, particularly propionic acid, which has been shown to activate hepatic MUP1 to alleviate endoplasmic reticulum stress and ultimately ameliorate ALD. The authors further demonstrated that the health‐benefiting effects of urolithin A on ALD were microbiota‐dependent, as the depletion of gut microbiota using antibiotics attenuated these effects. These findings emphasize the crucial role of the intestinal microbiota in mediating the protective effects of urolithin A against ALD.

Notably, urolithin A has also been shown to alleviate metabolic disorders and chronic diseases independent of the intestinal microbiota. For example, in a study by Xiao et al. (2022), urolithin A (200 mg kg^−1^) attenuated diabetes‐related cognitive impairment in high‐fat diet and streptozotocin‐induced type 2 diabetes mellitus mice via restoring gut barrier through promoting N‐glycan biosynthesis. The authors found that urolithin A ameliorated the hyperglycemia‐associated reduced expression of N‐glycan biosynthesis‐involved genes, which are crucial for intestinal barrier integrity. Additionally, urolithin A reduced the concentrations of metabolic endotoxemia and proinflammatory cytokines, suggesting a systemic anti‐inflammatory impact. However, urolithin A had no significant modulating effect on gut microbiota and SCFAs. These findings suggested that the effect of urolithin A was independent of the gut microbiome.

In conclusion, EA and its urolithin metabolites have a profound impact on the intestinal microbiota, affecting both microbial community composition and functional profile. These changes are closely associated with improved intestinal health and systemic benefits, highlighting the potential of EA and urolithins as dietary interventions to enhance gut health and prevent gastrointestinal disorders. However, further research is necessary to comprehensively elucidate the precise mechanisms underlying the effects of EA and its urolithin metabolites on the intestinal microbiota, determine optimal supplementation dosages, and evaluate their effects across diverse populations and conditions.

## 4 Health Benefits of EA and Its Intestinal Microbial Metabolites Urolithins

EA is a natural polyphenolic compound found in various plant‐based foods such as pomegranates, berries, and nuts. Despite its potent bioactivities, the low bioavailability of EA, with a maximum concentration of 120 ng/mL in human plasma, limits its systemic effects in vivo (Kang et al. 2016; Alfei et al. 2020). After ingestion, EA is metabolized by the gut microbiota into urolithins and dibenzopyran‐6‐one derivatives with varying hydroxyl substitutions and greater lipophilicity, demonstrating 25–80‐fold higher bioavailability and superior absorption compared to EA, and exhibiting unique bioactivities (Alfei et al. 2020; Lin et al. 2023). This section compares the bioactivities of EA and its urolithin metabolites, highlighting their shared and unique properties.

### 4.1 Antioxidant

Ellagic acid exhibits multifunctional antioxidant properties both in vitro and in vivo. Studies using ferrous ion oxidation, glutathione quantification, and 2′,7′‐dichlorofluorescin diacetate staining confirmed the capacity of EA to attenuate H_2_O_2_ exposure‐induced oxidative stress and enhance intracellular antioxidant defenses rather than pro‐oxidant activity (Weisburg et al. 2013). DPPH, FRAP, NO, and superoxide scavenging assays consistently demonstrated that EA possessed strong antioxidant capacity (Verotta et al. 2018). EA reduced cellular reactive oxygen species (ROS) levels, thereby protecting cells from mitochondrial dysfunction, apoptosis, and necrosis induced by oxidative stress (Ding et al. 2014). In V79‐4 cells, EA treatment significantly elevated the antioxidant enzymatic activities, such as glutathione peroxidase (GPX), catalase (CAT), and superoxide dismutase (SOD), and exhibited considerable lipid peroxidation inhibiting and DPPH radical scavenging activities. Additionally, EA showed antioxidant bioactivity by upregulating Bax and activating caspase‐3 (Han et al. 2006).

Experimental evidence demonstrates that EA primarily exerts its antioxidant effects via NF‐κB and Nrf2 pathways. The Keap‐1‐Nrf2 pathway mechanism regulates the balance between oxidative and antioxidative states in the body. The Keap1‐Nrf2 pathway is crucial in regulating redox homeostasis. Under basal conditions, Nrf2 is sequestered in the cytoplasm by Keap1; upon activation, it translocates to the nucleus to initiate antioxidant gene transcription. EA reduced ROS production via the miR‐223/Keap1/Nrf2 pathway, downregulated malondialdehyde (MDA) generation, and upregulated SOD activity, thereby improving oxidative stress and alleviating insulin resistance in HepG2 cells treated with high glucose (Ding et al. 2019). Activation of the Keap‐1‐Nrf2 pathway increases the expression of downstream enzyme genes, including heme oxygenase‐1 (HO‐1) and SOD. EA enhanced the expression of antioxidant enzymes SOD and HO‐1 in human keratinocytes via the Keap1‐Nrf2 system, thereby mitigating oxidative harm to HaCaT cells induced by UVA radiation (Hseu et al. 2012). EA activated Nrf2 migration to the nucleus and inhibited NF‐κB signaling, increasing the expression of HO‐1 and SOD, thus providing neuroprotection (Li et al. 2017). EA had the capacity to inhibit NF‐κB activation, reducing oxidative stress and inflammatory responses, thus protecting Wistar rats from cyclophosphamide‐induced acute lung injury (Raisuddin and Beigh 2013). EA safeguarded male rats against oxidative stress‐related damage to the testes and sperm induced by cyclosporine by enhancing antioxidant enzyme activities and inhibiting lipid peroxidation. Furthermore, the sperm concentration in the epididymis of rats treated with EA was notably greater than that observed in the model group (Türk et al. 2010). Oral EA supplement prevented isoproterenol‐induced myocardial infarction oxidative stress and restored arterial blood pressure and heart rate in rats with myocardial infarction (Kannan and Quine 2011).

Ellagic acid is a potent antioxidant due to its polyphenolic structure, which allows it to directly scavenge free radicals and participate in redox reactions. Studies have demonstrated that EA effectively reduces oxidative stress in vitro and in localized tissues, such as the gastrointestinal tract. However, its poor solubility and limited absorption constrain its systemic antioxidant effects. In contrast, urolithins exhibit antioxidant activity primarily through indirect mechanisms. Urolithin A, the most studied metabolite, has been shown to upregulate endogenous antioxidant pathways such as the Nrf2 signaling pathway (Espín et al. 2013). While urolithins have a lower intrinsic radical‐scavenging ability than EA, their higher bioavailability enables them to exert systemic antioxidant effects in vivo.

Urolithins share antioxidant mechanisms with EA, primarily via the Nrf2 and NF‐κB pathways. In polyinosinic‐polycytidylic acid (poly(I:C))‐induced RAW264.7 macrophages (a TLR3 agonist that triggers macrophage inflammation), urolithin A suppressed TLR3‐mediated oxidative‐inflammatory crosstalk by inhibiting NF‐κB and ERK/mitogen‐activated protein kinase (MAPK) signaling pathways, while enhancing CAT and SOD activities and reducing MDA levels. It demonstrated that urolithin A suppressed the oxidative and inflammatory pathways activated by TLR3 in macrophages (Huang et al. 2022). Similarly, urolithin B showed antioxidant effects by reducing ROS and the expression of NADPH oxidase subunits and elevating the expression of antioxidant HO‐1 through the Nrf2/ARE pathway. Urolithin B exerted its antioxidant effects by inhibiting NF‐κB activity and decreasing the phosphorylation and degradation of IκBα (Lee et al. 2019).

Notably, EA and urolithins can exhibit opposing effects on oxidative stress. In all‐trans retinoic acid (ATRA)‐treated U937 cells, EA (20 μM) inhibited superoxide anion generation by suppressing gp91‐phox transcription, histone H3‐Lys9 acetylation at the gp91‐phox promoter, and cytochrome b558 components (p22‐phox/gp91‐phox). Conversely, urolithin A (20 μM) enhanced these processes, amplifying superoxide production (Kikuchi et al. 2021). These findings suggest that conversion of EA to urolithin A in the gut might lead to contrasting physiological outcomes related to oxidative stress.

### 4.2 Anti‐Inflammatory

Inflammation is a chronic symptom associated with various diseases and injuries, posing significant harm to the body. In addition to their well‐known antioxidant effects, EA and urolithins have been reported to possess effective anti‐inflammation activities. The anti‐inflammatory and antioxidant mechanisms of EA and its metabolites often share mechanisms with their antioxidant actions, involving tightly connected pathways.

EA alleviated IL‐1β‐induced inflammation in human chondrocytes by inhibiting the expression of matrix metalloproteinase‐13 (MMP‐13), cyclooxygenase‐2 (COX‐2), a disintegrin and metalloproteinase with thrombospondin motifs 5 (ADAMTS5), and inducible nitric oxide synthase (iNOS), and reducing the generation of NO, prostaglandin E2 (PGE2), IL‐6, and TNF‐α (Haidari et al. 2009). EA significantly downregulated the concentrations of hepatic cytokines, including IL‐6, IL‐1β, TNF‐α, NF‐κB, and IL‐10, potentially through the LPS‐TLR4‐NF‐κB signaling pathway. EA significantly increased cellular levels of IκB, suppressing NF‐κB expression in a dose‐dependent manner. EA suppressed LPS‐induced inflammation in RAW264.7 macrophages by inhibiting MAPK, ERK, JNK, and p38 activation. Studies using TLR4/MD‐2‐specific antibodies indicated that EA primarily targets the TLR4/MD‐2 complex (Du et al. 2019). In a DSS‐induced rat model of chronic colitis, dietary EA intake inhibited the phosphorylation of p38 and MAPK and suppressed NF‐κB‐mediated transcriptional activation (Marín et al. 2013). EA also showed promising therapeutic effects in rheumatoid arthritis models. In the TNF‐α‐induced MH7A human fibroblast‐like synoviocyte inflammation model, EA intervention reduced MTA1 protein expression, reduced the activity of the MTA1/HDAC1 complex in MH7A cells, promoted apoptosis, and suppressed inflammation. EA alleviated the arthritis index, synovial hyperplasia, paw swelling, and inflammatory response in rats with collagen‐induced arthritis, thereby mitigating the severity of rheumatoid arthritis (Song et al. 2021).

Similar to EA, urolithin A inhibited TLR3 expression by suppressing IRF3 phosphorylation and reducing IFN‐α/β secretion. Additionally, urolithin A suppressed NF‐κB/STAT1 and ERK/MAPK signaling cascades, thereby reducing levels of inflammatory mediators, such as MCP‐1, CCL‐5, TNF‐α, and iNOS, in macrophages stimulated by polyinosinic‐polycytidylic acid. The results indicated that urolithin A suppressed inflammatory response pathways activated by TLR3 in macrophages, explaining its anti‐inflammatory effects (Huang et al. 2022). Urolithin B inhibited the generation of pro‐inflammatory cytokines and NO, and increased IL‐10, an anti‐inflammatory cytokine, in LPS‐stimulated BV2 microglial cells. Beyond LPS stimulation, urolithin B inhibited IL‐6, TNF‐α, and NO production in BV2 cells stimulated by polyinosinic‐polycytidylic acid or lipoteichoic acid. Urolithin B inhibited ERK, JNK, and Akt phosphorylation, while enhancing AMPK phosphorylation, which linked its anti‐inflammatory effects to its antioxidant mechanisms (Lee et al. 2019).

### 4.3 Anticancer

EA and its metabolites, urolithins, have demonstrated significant anticancer potential by enhancing survival rates, reducing mortality, and selectively targeting cancer cells without harming normal cells. In a study on Caco‐2 human colon cancer cells and CCD‐112CoN normal colon cells, EA caused cell cycle arrest at the S phase by reducing cyclins A and B1 and increasing cyclin E levels. It triggered apoptosis via intrinsic pathways, as evidenced by reduced bcl‐XL expression, mitochondrial cytochrome c release, and activation of caspases 9 and 3. Notably, normal CCD‐112CoN cells did not exhibit chromatin condensation or caspase activation, highlighting the selective cytotoxicity of EA. EA induced apoptosis through the mitochondrial pathway in cancer cells, sparing normal cells (Larrosa et al. 2006). The anticancer activity of EA has also been explored in oral cancer. In HSC‐2 oral cancer cells, EA induced apoptosis, as observed through apoptotic morphology, increased caspase‐3/7 activity, and cleavage of poly ADP‐ribose polymerase (PARP). Importantly, EA selectively targeted cancer cells while sparing normal gingival fibroblasts, underscoring its therapeutic potential (Weisburg et al. 2013). EA demonstrates multifaceted anticancer effects across gastrointestinal (GI) cancers by targeting key molecular pathways, including antioxidant/anti‐inflammatory actions, cell cycle arrest, mitochondrial apoptosis induction, and inhibition of survival pathways. In esophageal cancer, EA inhibits carcinogen‐induced DNA adducts and suppresses STAT3 signaling while stabilizing tumor suppressor proteins. In gastric cancer, EA modulates p53, PI3K‐Akt, and COX‐1/2 inflammatory pathways, reducing proliferation, invasion, and *Helicobacter pylori* activity. In colorectal cancer, EA induces apoptosis via caspase activation, inhibits Wnt/β‐catenin and PI3K/Akt pathways, and enhances chemosensitivity to 5‐fluorouracil. Pancreatic cancer studies show EA suppresses NF‐κB/COX‐2, triggers caspase‐3/9‐mediated apoptosis, and inhibits epithelial‐mesenchymal transition by regulating E‐cadherin and MMPs (Chauhan et al. 2024).

Urolithin A, a major metabolite of EA, exhibited potent anticancer effects, particularly in colorectal and oral cancers. At micromolar concentrations, urolithin A induces apoptosis and autophagy, inhibits the advancement of the cell cycle, and suppresses DNA synthesis. In SW620 colorectal cancer (CRC) cells, sub‐micromolar levels of urolithin A induced autophagy, a conserved biological process involving the degradation and recycling of cytoplasmic components, thus suppressing the survival and metastasis of SW620 cells. Additionally, urolithin A exposure decreased cell growth, delayed cell migration, and reduced MMP‐9 activity in a dose‐dependent manner, thereby reducing cancer invasiveness and metastasis. These findings indicated that urolithin A stimulated autophagy and impeded the metastasis of human CRC cells (Zhao et al. 2018). In oral squamous cell carcinoma (OSCC), urolithin A significantly promoted cancer cell death by triggering endoplasmic reticulum stress, which subsequently led to the inhibition of the AKT and mTOR pathways. Additionally, evidence from in vitro and in vivo studies revealed urolithin A‐induced apoptosis and autophagy, reducing tumor volume and proliferation markers like Ki67. These effects were mediated through the inhibition of AKT/mTOR/ERK signaling, highlighting its dual role in inducing cell death and inhibiting tumor growth (Remadevi et al. 2024). Urolithin A exhibits good safety and is applicable for both the prevention and treatment of cancer. Urolithin A reduced the generation of pro‐inflammatory factors, such as IL‐6, IL‐1β, and NOS2. Oral intake of urolithin A displayed significant anticancer and anti‐inflammatory impacts in various in vivo models, including colitis, carrageenan‐induced paw edema, obesity, and pancreatic cancer (Rogovskii 2022). The primary molecular mechanisms of these effects are likely related to the regulation of the aryl hydrocarbon receptor, reduced levels of protein kinase B phosphorylation, and stabilization of p53. In prostate cancer, urolithin A showed chemopreventive and therapeutic efficacy. It significantly inhibited the growth of C4‐2B xenografts, downregulated androgen receptor expression, suppressed cell proliferation, and reduced AKT phosphorylation levels, thereby exerting therapeutic effects against prostate cancer (Ding et al. 2020).

In an antiproliferative assay against human bladder cancer cell lines T24, EA, urolithin A, urolithin B, and 8‐OMe‐urolithin A inhibited cell growth, with the IC 50 values of 33.7, 43.9, 35.2, and 46.3 μM, respectively. Urolithins, especially urolithin B, exhibited inhibitory potential comparable to EA. EA and urolithins activated p38 MAPK while inhibiting MEKK1 and c‐Jun, leading to caspase‐3 activation and apoptosis, suggesting a shared mechanism of action in inducing apoptosis and contributing to their antitumor effects (Qiu et al. 2013).

Notably, EA exhibited distinct effects compared to urolithins in some cases. EA (300 μg/mL) significantly promoted PC12 cell proliferation, and urolithin A, B, and C significantly inhibited PC12 cell proliferation at higher concentrations, with urolithin C exhibiting the most pronounced inhibitory effect. Urolithin C treatment significantly increased lactate dehydrogenase (LDH) release and MDA levels, stimulated ROS formation, led to mitochondrial membrane depolarization, and caused calcium dyshomeostasis. Urolithin C treatment induced apoptosis and S‐phase cell cycle arrest, with the proportion of apoptotic cells increasing significantly with increasing urolithin C concentrations. Urolithin C treatment resulted in an imbalance in the Bcl‐2/Bax ratio, triggering the caspase cascade and thus promoting apoptosis. Urolithin C treatment decreased the Bcl‐2/Bax protein and mRNA ratios and increased the expression of caspase‐3 and caspase‐9 mRNA. Compared to EA, urolithins, especially urolithin C, exhibited significant antiproliferative activity and cytotoxicity in PC12 cells, inducing apoptosis through a mitochondria‐mediated pathway. These findings not only broaden our understanding of the biological activities of EA gut metabolites but also provide a basis for the future development of urolithin C as a novel antitumor agent (Yin et al. 2017). Urolithins exhibit excellent safety profiles, making them suitable for both cancer prevention and treatment. Their multifaceted effects on inflammation, oxidative stress, and tumor growth suggest they are promising candidates for future clinical applications.

### 4.4 Intestinal Barrier Function

EA has been reported to protect intestinal barrier function in various in vivo and in vitro studies. Dietary EA supplementation significantly decreased serum diamine oxidase and LPS levels and increased mRNA expression of claudin‐1 in the jejunum, ZO‐1 and claudin‐1 in the ileum of the heat‐stressed broilers (Yang et al. 2022). EA supplementation restored the TNBS‐induced reduction in tight junction proteins via regulating the RhoA/ROCK/MLC signaling pathway in SD rats, maintaining intestinal barrier function (Peng et al. 2023). EA mitigated TNFα‐triggered Caco‐2 monolayer permeabilization through NF ‐KB, ERK1/2, and MLCK inhibition (Iglesias et al. 2020).

Urolithin A enhanced intestinal barrier integrity through dual mechanisms involving direct cellular signaling and microbiota modulation. In conventional and colitis‐induced mice, oral administration of urolithin A strengthened the mucus layer by activating the aryl hydrocarbon receptor (AhR) and Nrf2 pathways, upregulating mucin 2 production in goblet cells, which reduced intestinal permeability and alleviated colitis severity. Concurrently, urolithin A enriched SCFA‐producing bacteria (e.g., *Ruminococcus*, *Prevotella*) and elevated fecal propionate levels, indirectly supporting barrier function through microbial metabolite‐driven pathways. These combined actions synergistically protect against inflammation, improve mucosal integrity, and mitigate colitis, highlighting the therapeutic potential of urolithin A for gut barrier disorders (Yasuda et al. 2024). Urolithin A protected intestinal barrier function by mitigating oxidative stress and inflammation induced by environmental toxins like inorganic arsenic (iAs^3+^). In colon epithelial cells and 3D human intestinal organoids, urolithin A reduced iAs^3+^‐induced cytotoxicity, apoptosis, and ROS generation while restoring glutathione levels to counteract oxidative damage. It enhanced gut barrier integrity by preserving tight junction proteins (ZO‐1, occludin, claudin‐4) through mechanisms involving AhR‐Nrf2 pathway activation, which promoted antioxidant responses and suppresses pro‐inflammatory cytokines (e.g., IL‐8, TNF‐α). Urolithin A also reduced intestinal permeability and maintained trans‐epithelial electrical resistance, demonstrating its potential to counteract environmental toxin‐induced barrier dysfunction by reinforcing tight junction stability and mitigating oxidative and inflammatory stress (Ghosh et al. 2022). Urolithin A could also attenuate diabetes‐associated intestinal barrier dysfunction via the N‐glycan biosynthesis pathway (Xiao et al. 2022).

Ellagic acid and urolithin A showed different protective effects on intestinal barrier function through distinct mechanisms in vitro. EA strengthened the ileal barrier in Caco‐2 cells by suppressing pore‐forming claudin−4, −7, and −15 via MLCK‐mediated MLC2 signaling, resulting in increased transepithelial resistance (TER) and reduced small‐molecule permeability. In contrast, urolithin A counteracted TNFα‐induced colonic barrier damage in HT‐29/B6 cells by preventing inflammation‐driven upregulation of sealing claudin−1 and −2, and their pathological redistribution, partially reversing TER decline. While EA acted preventively through structural reinforcement without direct anti‐inflammatory effects, urolithin A specifically targeted inflammatory barrier disruption. Both compounds demonstrated compartment‐specific efficacy, EA in ileum‐like models and urolithin A in colonic systems, highlighting their complementary therapeutic potential for gut disorders (Hering et al. 2021).

### 4.5 Metabolic and Cardiovascular Effects

Ellagic acid and urolithins demonstrated distinct antiatherogenic effects. Both EA and urolithins exhibited anti‐inflammatory effects by reducing monocyte adhesion and cytokine secretion. However, urolithin C and the combination of urolithins A and B were more effective in reducing monocyte adhesion compared to EA alone. EA and urolithins also reduced cholesterol uptake by macrophages. EA showed consistent effects across different cholesterol sources, while urolithin C and the combination of A and B were effective against hypercholesterolemic serum but not acetylated LDL. Neither EA nor urolithins significantly promoted cholesterol efflux from macrophages. Both EA and urolithins underwent extensive metabolism in peripheral cells, involving sulfate and methyl conjugation. However, the specific metabolites formed and their biological activities could differ. Collectively, both EA and urolithins exhibit anti‐atherogenic potential by modulating monocyte adhesion, cytokine secretion, and cholesterol uptake. However, urolithin C and the combination of urolithins A and B showed superior anti‐inflammatory effects compared to EA alone, suggesting that gut microbiota‐derived urolithins may play a significant role in the cardioprotective effects of ellagitannin‐rich foods (Mele et al. 2016). Urolithin A also demonstrated significant anti‐obesity effects by increasing energy expenditure through brown adipose tissue activation and white adipose tissue browning in mice. Urolithin A elevated triiodothyronine levels in adipose tissues by upregulating deiodinase 2, thereby stimulating thermogenic gene expression, including UCP‐1, PGC1α, and mitochondrial biogenesis (Xia et al. 2020).

### 4.6 Other Bioactivities

Ellagic acid and urolithins are expected to show endocrine effects due to their chemical structures. Skledar et al. tested their effects on estrogen, androgen, glucocorticoid, and thyroid hormone receptors in vitro using reporter gene cell line assays. EA and urolithin D did not show estrogenic agonist activity in the tested conditions, and urolithins A and B demonstrated estrogenic agonist activities on the estrogen receptor subtype α, with EC 50 values of 5.59 μM and 32.60 μM, respectively. EA, urolithin A, and urolithin D exhibited anti‐thyroid hormonal activity with EC50 values of 37.45 μM, 30.32 μM and 8.80 μM, respectively. Urolithin B did not show significant anti‐thyroid hormonal activity. EA and urolithins A, B, and D exhibited weak or no glucocorticoid or androgen agonist or antagonist activities (Skledar et al. 2019). These findings revealed notable differences in the bioactivities of EA and urolithins and highlighted the diverse and sometimes contrasting endocrine effects of these compounds, warranting further investigation into their potential health impacts and risks, especially in relation to human exposure.

Urolithins differentially affect the growth, adhesion, motility, and invasion of endometriotic cells. Urolithin A and urolithin B exhibited inhibitory effects on the proliferation of endometriotic cells, but urolithin A was more potent. In St‐T1b cells, the IC50 value of urolithin A was 39.88 μM, while that of urolithin B was 79.92 μM. Both urolithin A and urolithin B increased the proportion of S‐phase cells in St‐T1b cells, suggesting that they inhibit cell proliferation by affecting the cell cycle. However, in 12Z cells, only urolithin A increased the proportion of G2/M‐phase cells. Urolithin A significantly increased the apoptosis rate of St‐T1b cells, while urolithin B had no such effect. This indicated that urolithin A had unique activity in inducing cell apoptosis. Urolithin B significantly reduced the adhesion ability of 12Z cells, while urolithin A had no such effect. This suggested that urolithin B had a specific role in regulating cell adhesion. Both urolithin A and urolithin B significantly reduced the migration and invasion abilities of 12Z cells. However, in terms of migration, urolithin A had a more pronounced inhibitory effect on St‐T1b cells. Urolithin A significantly downregulated the expression of MMP2 in 12Z cells, while both urolithin A and urolithin B downregulated the expression of MMP3 and MMP9. In addition, urolithin A downregulated the expression of ROCK2, while urolithin B downregulated RAC1. These changes may explain their inhibitory effects on cell migration and invasion. In three‐dimensional co‐culture spheroid and organoid models, both urolithin A and urolithin B significantly reduced the viability and integrity of spheroids and organoids. However, urolithin A usually exhibited more potent inhibitory effects (Mc Cormack et al. 2021).

Ellagic acid and urolithins exhibit distinct effects on circadian rhythm regulation. In senescent human fibroblasts, EA showed no significant impact on circadian clock gene oscillations, whereas urolithin A prominently enhanced the amplitude of *Bmal1*‐driven luciferase rhythms without altering period length. Urolithin A achieved this by destabilizing PER2 protein via SIRT1‐mediated deacetylation, which promotes PER2 degradation and strengthens CLOCK/BMAL1 transcriptional activity, thereby amplifying circadian output (Kuatov et al. 2025). In inflammatory bowel models, urolithin A improved circadian dysregulation by the restoring rhythmic expression of clock genes (BMAL1, PER2) and tight junction proteins in intestinal epithelial cells. This effect involved the Nrf2‐SIRT1 pathway, with urolithin A pretreatment mitigating colitis‐induced disruptions in fecal IgA rhythms and central clock (SCN) synchronization (Du et al. 2024). These studies demonstrated the role of urolithin A in modulating circadian rhythms through SIRT1, and no direct circadian effects of EA were observed, which underscored the superior bioactivity of urolithin A as a gut microbiota‐derived metabolite, particularly in aging and inflammatory contexts where circadian robustness is compromised.

Urolithin A showed improvements in mitochondrial function and contributed to derived health benefits, such as muscle performance and age‐related tissue health across preclinical and clinical studies (D'Amico et al. 2021). Urolithin A enhances mitochondrial quality by stimulating mitophagy, the selective degradation of dysfunctional mitochondria, leading to improved skeletal muscle endurance and strength. In older adults, 4‐month urolithin A supplementation (1000 mg/day) increased muscle endurance in hand and leg muscles by 95% and 41%, respectively, compared to placebo, while reducing plasma biomarkers of mitochondrial dysfunction (acylcarnitines and ceramides) and inflammation (Liu, D'Amico, et al. 2022). Similarly, in middle‐aged adults, urolithin A improved muscle strength (~12% in hamstrings) and aerobic capacity, with increased expression of mitophagy‐related proteins, including PINK1 and Parkin, in skeletal muscle (Singh et al. 2022). Urolithin A enhanced mitochondrial respiration in human chondrocytes and reduced osteoarthritis progression in mice by upregulating mitophagy flux and mitochondrial content in joint tissues (D'Amico et al. 2022). In *C. elegans* , urolithin A extended lifespan by 45% and preserved mobility through mitophagy‐dependent maintenance of mitochondrial function, while EA showed no such effects (Ryu et al. 2016). Notably, the benefits of urolithin A were distinct from its precursor EA since EA lacked direct mitophagy‐inducing activity. Unlike urolithin A, EA failed to improve lifespan or mitochondrial parameters in worms and required microbial conversion to bioactive urolithins for efficacy (Ryu et al. 2016; Liu, D'Amico, et al. 2022). These findings suggest urolithin A as a postbiotic therapeutic candidate for mitigating age‐related declines in muscle, joint, and mitochondrial health.

## 5 Conclusions and Perspectives

In conclusion, the interactions between EA and intestinal microbiota have profound implications for human health. The research summarized in this review demonstrates that EA can positively modulate intestinal microbiota, improve the generation of short‐chain fatty acids, and enhance gut barrier function. These effects play a critical role in mitigating metabolic and chronic illnesses. Additionally, EA and its gut‐derived metabolites demonstrate antioxidant, anti‐inflammatory, anti‐cancer, and other health‐benefiting properties, underscoring their potential as therapeutic agents.

Looking forward, several key research directions merit attention. A deeper understanding of the mechanisms governing EA interaction with the intestinal microbiota is essential. Future research should focus on elucidating the metabolic pathways of EA in the gut and characterizing the microbiota compositions and functions associated with varying metabolic phenotypes. In addition, the diverse pharmacological activities of EA and its metabolites present opportunities for developing innovative therapeutic agents. Further studies are needed to explore their potential in preventing and treating diseases, including cancer, inflammatory disorders, and metabolic syndromes. EA and its microbial metabolites could serve as the basis for new drugs or functional foods aimed at improving human health. Overall, the dynamic interplay between EA and intestinal microbiota represents a promising frontier in health science. Continued research in this area will not only deepen our understanding of the health benefits of EA but also pave the way for novel interventions to enhance human well‐being.

## Author Contributions

**Pinze Leng:** conceptualization (supporting), investigation (lead), writing – original draft (lead). **Ye Wang:** investigation (supporting), writing – original draft (supporting), writing – review and editing (supporting). **Minhao Xie:** conceptualization (lead), funding acquisition (lead), supervision (lead), writing – review and editing (lead).

## Conflicts of Interest

The authors declare no conflicts of interest.

## Data Availability

The authors have nothing to report.
